# Daily zeaxanthin supplementation prevents atrophy of the retinal pigment epithelium (RPE) in a mouse model of mitochondrial oxidative stress

**DOI:** 10.1371/journal.pone.0203816

**Published:** 2018-09-28

**Authors:** Manas R. Biswal, Bradley D. Justis, Pingyang Han, Hong Li, Dennis Gierhart, Cheryl K. Dorey, Alfred S. Lewin

**Affiliations:** 1 Department of Molecular Genetics and Microbiology, College of Medicine, University of Florida, Gainesville, FL, United States of America; 2 Center for Vision Research, University of Florida, Gainesville, FL, United States of America; 3 College of Pharmacy, University of South Florida, Tampa, United States of America; 4 ZeaVision, Chesterfield, MO, United States of America; 5 Virginia Tech Carilion School of Medicine, Roanoke, Virginia, United States of America; 6 Department of Ophthalmology, College of Medicine, University of Florida, Gainesville, FL, United States of America; University of Manchester, UNITED KINGDOM

## Abstract

Oxidative damage is implicated in the pathogenesis of age-related macular degeneration (AMD). The dry form of AMD (geographic atrophy) is characterized by loss of RPE, photoreceptors, and macular pigments. The cumulative effects of oxidative stress impact mitochondrial function in RPE. In *Sod2*^*flox/flox*^*VMD2-cre* mice, the RPE specific deletion of *Sod2*, the gene for mitochondrial manganese superoxide dismutase (MnSOD), leads to elevated oxidative stress in retina and RPE, and causes changes in the RPE and underlying Bruch’s membrane that share some features of AMD. This study tested the hypothesis that zeaxanthin supplementation would reduce oxidative stress and preserve RPE structure and function in these mice. Zeaxanthin in retina/RPE/choroid and liver was quantified by LC/MS, retinal function and structure were evaluated by electroretinogram (ERG) and spectral domain optical coherence tomography (SD-OCT), and antioxidant gene expression was measured by RT-PCR. After one month of supplementation, zeaxanthin levels were 5-fold higher in the retina/RPE/choroid and 12-fold higher in liver than in unsupplemented control mice. After four months of supplementation, amplitudes of the ERG a-wave (function of rod photoreceptors) and b-wave (function of the inner retina) were not different in supplemented and control mice. In contrast, the c-wave amplitude (a measure of RPE function) was 28% higher in supplemented mice than in control mice. Higher RPE/choroid expression of antioxidant genes (*Cat*, *Gstm1*, *Hmox1*, *Nqo1*) and scaffolding protein *Sqstm1* were found in supplemented mice than in unsupplemented controls. Reduced nitrotyrosine content in the RPE/choroid was demonstrated by ELISA. Preliminary assessment of retinal ultrastructure indicated that supplementation supported better preservation of RPE structure with more compact basal infoldings and intact mitochondria. We conclude that daily zeaxanthin supplementation protected RPE cells from mitochondrial oxidative stress associated with deficiency in the MnSOD and thereby improved RPE function early in the disease course.

## Introduction

Age-related macular degeneration (AMD) is a leading causes of irreversible vision loss among older adults. More than 11 million American have some form of AMD, and this number is expected to increase substantially by 2050 [1]. Advanced AMD is typically manifested as choroidal neovascularization (wet AMD) or as geographic atrophy, referring to the progressive death of RPE and photoreceptor cells in the macula (dry AMD) [2], both types cause significant loss of vision. Treatments targeting vascular endothelial growth factor (VEGF) are available for neovascular AMD, but effective treatment for geographic atrophy is urgently needed.

Retinal pigment epithelial (RPE) cells provide essential functions to support photoreceptors, including transport of nutrients and oxygen from the choroid to photoreceptors, phagocytosis of photoreceptor outer segments, and recycling of 11-cis-retinal, as part of the visual cycle [3]. Early AMD is characterized by drusen—yellow deposits of oxidized lipids and protein that lie beneath the RPE layer in the macular region. Geographic atrophy in advanced dry-AMD is a well-defined progressively enlarging area where photoreceptors, RPE and the underlying choriocapillaris have been lost. While genetic risk factors for AMD connect dysregulation of the alternative complement cascade with AMD [4], the presence of oxidatively damaged biomolecules in the RPE from AMD eyes [5–7] and ability of antioxidants in the AREDS formulas to reduce risk for progression to advanced AMD [8] implicate oxidative stress as a contributing factor. In fact, oxidized lipids and fragments of oxidized docashexaenoates activate complement and induce neovascularization in animal models [9–11]. Oxygen consumption by the central retina is very high, and the metabolic demands necessary for the RPE to serve photoreceptor needs are also substantial [12]. Under normal conditions, mitochondrial electron transport generates reactive oxygen species at complexes I and III, and superoxide production may increase with age[13]. MnSOD in the mitochondria protects against oxidative damage to critical molecules [14–15].

The importance of oxidative stress in this disease is reflected by the fact that smoking is secondary only to age as a risk factor for AMD [16]. Another indication that oxidative stress contributes to AMD comes from finding that dietary intervention with antioxidants retards the progression of the disease. The Age-Related Eye Disease Study (AREDS) formulation (which contains vitamins C and E, beta-carotene and zinc) lowered risk of progression to advanced neovascular AMD, confirming the role of oxidative stress in AMD [17–18]. Of the 20+ carotenoids in the diet, only two, lutein and zeaxanthin are accumulated in the retina as the macular pigment. Meso-zeaxanthin, an isomeric metabolite of lutein, also accumulates [19–23]. Xanthophylls (lutein and zeaxanthin) and n–3 fatty acids are essential for the development and maintenance of a normal distribution of RPE cells [24]. AREDS II determined that anti-oxidant supplements including lutein and zeaxanthin reduced progression to advanced AMD with choroidal neovascularization and that lutein and zeaxanthin may be superior to supplements containing beta-carotene, because of the increased cancer risk of beta-carotene among smokers [8, 25]. Reduced risk for progression to geographic atrophy has been associated with greater consumption of omega-3 long-chain polyunsaturated fatty acids [26], which are essential for the photoreceptors.

Consequently, supplementing the diet with higher levels of dietary lutein or zeaxanthin to improve antioxidant protection in RPE could be a strategy to reduce or prevent progression of early AMD to geographic atrophy. Zeaxanthin, a plant-derived antioxidant has been shown to decrease oxidative stress markers and enhance antioxidant capacity in cultured cells [27]. Dorey *et al*., found that zeaxanthin supplementation produced a dose-dependent increase in retinal zeaxanthin, and prevented light-induced photoreceptor cell death in quail [28–30]. Several studies have shown antioxidants protect photoreceptors *in vivo*[31] and RPE cells *in vitro* [32–33]. Zeaxanthin and vitamin E protect phagocytic activity in RPE cells by reducing reactive oxygen species caused by light-irradiated lipofuscin [34]. We previously reported that elevated mitochondrial oxidative stress caused RPE dysfunction, damage to the choroid, and death of photoreceptor cells in a mouse model [35]. We therefore hypothesized that dietary zeaxanthin would modulate RPE atrophy due to oxidative stress resulting from MnSOD deletion in this model.

In this study, we tested the potential for daily zeaxanthin supplements to protect RPE cells in *Sod2*^flox/flox^*VMD2-cre* mice in which the RPE-specific deletion of *Sod2*—the gene for manganese superoxide dismutase (MnSOD)—leads to elevated oxidative stress and RPE dysfunction [35]. We detected increased zeaxanthin in retina/RPE/choroid and liver, increased expression of antioxidant genes of the *Nrf2* pathway in the retina/RPE, reduced RPE thinning and preserved RPE function compared with unsupplemented *Sod2*^*flox/flox*^*VMD2-cre* mice. Our results indicate that dietary zeaxanthin supplementation prevents oxidative stress in RPE, identify zeaxanthin supplementation as a way to protect RPE functions and point to its possible potential to reduce the risk for progression to dry AMD.

## Materials and methods

### Animals

All experimental procedures involving animals were performed according to the ARVO Statement for the Use of Animals in Ophthalmic and Vision Research with the approval of the Institutional Animal Care and Use Committee at the University of Florida. *Sod2*^*flox/flox*^*-VMD2-cre* mice were transgenic for PVMD2-rtTA and tetO-PhCMV *cre* and were homozygous for *Sod2* containing loxP sites surrounding exon 3, which encodes the manganese binding site of superoxide dismutase. In these mice, only treatment with doxycycline activates *cre* recombinase and that leads to *Sod2* deletion only in the RPE due to cre expression driven by the RPE specific VMD2 promoter [36]. Two month old (male and female) mice were chosen for zeaxanthin treatment experiments. The mice were housed in a controlled environment at 20–24°C under pathogen-free conditions with a 12-h light-dark cycle and ad libitum access to food and water. Expression of the cre transgene was induced in neonatal mice by feeding nursing dams doxycycline-containing chow (200 mg/kg rodent diet [T-7012, 200 doxycycline]; Harlan Laboratories, Inc., Tampa, FL, USA) for 2 weeks immediately after the birth of the litter [35]. Importantly, doxycycline treatment was completed more than three months before measurement of oxidative injury or retinal function.

### Zeaxanthin treatment

Zeaxanthin encapsulated beadlets having 5% zeaxanthin were provided by EyePromise (Chesterfield, MO). These beadlets were mixed with water at a concentration of 150 mg/ml (containing 7.5 mg/ml zeaxanthin), and 150μl to 200μl of the dissolved beadlets were delivered to 20 to 25 gm *Sod2*^*flox/flox*^*-VMD2-cre* mice (n = 12) for four months. Each mouse received a daily dosage of 55 to 60mg/kg of zeaxanthin through beadlets (1.125 mg of zeaxanthin for a 20gm mouse). A separate sham control group of *Sod2*^*flox/flox*^*-VMD2-cre* mice (n = 8) received no zeaxanthin and were housed with the treated mice and handled similarly on a daily basis. At the end of experiments, mice were euthanized either by inhalation of carbon dioxide (>90%) for biochemical studies or by injection of 150 mg/kg of sodium pentobarbital (as Euthasol™) for electron microscopy studies.

### Analysis of zeaxanthin concentration

Tissues were collected and snap frozen for zeaxanthin quantification by HPLC at Craft Technologies, Inc. (Wilson, NC) as described in detail by Toyoda *et al*. [30].

### Electroretinography

For electoretinography (ERG), mice were dark-adapted overnight. Prior to injection of anesthesia, the eyes were dilated twice (10–15 min apart) with one drop each of 2.5% phenylephrine hydrochloride ophthalmic solution (Paragon BioTek, Inc., Portland, OR) and 1% atropine sulfate ophthalmic solution (Akorn, Inc., Lake Forest, IL). The mice were anesthetized with a mixture of ketamine (95 mg/kg) and xylazine (5–10 mg/kg) by intraperitoneal injection. Prior to placing electrodes to record ERG, one drop of sterile lubricant eye drops (CVS Pharmacy, Inc., Woonsocket, RI) was applied to each of the eyes to prevent the eye from drying during procedures. To examine retinal responses, scotopic a-, b- and c-wave amplitudes from each eye were measured using Espion full-field ERG system (Diagnosys LLC, Lowell, MA 01851) at a flash intensity of 20 cds/m^2^) [37]; the results from treated and untreated eyes were compared.

### Spectral-Domain Optical Coherence Tomography (SD-OCT)

Eyes were dilated, and mice were anesthetized as described above for ERG analysis, High-resolution SD-OCT images were obtained by employing Envisu SD-OCT ophthalmic imaging system (Leica/Bioptigen, Durham, NC, USA) as described in our previous paper [38]. Photoreceptor survival was assessed by measuring the thicknesses of the outer nuclear layer (ONL) at four different locations (temporal, nasal, superior, and inferior) at 0.35-mm distance from the optic nerve head (ONH). Upon averaging the ONL thickness measurements from both the left and right eye, the results were compared between treated and untreated mice.

### Relative quantification of mRNA expression of antioxidant genes

The retina and RPE/choroid were dissected and collected in RNA *later* stabilization solution (ThermoScientific). Total RNA was isolated using the RNeasy mini kit (Qiagen), and first strand cDNA for each sample was generated using 500ng of total RNA (iScript from Biorad). Using universal SYBR Green Supermix (Biorad), primers for the mouse antioxidant genes catalase (*Cat*), glutathione S-transferase Mu 1 (*Gstm1*), heme oxygenase 1 (*Hmox1*), NAD(P)H quinone dehydrogenase 1 (*Nqo1*), and sequestosome 1 (*Sqstm1*) were designed using the NCBI primer database to amplify 200bp amplicons for each gene (S1 Table). Real Time-PCR amplification was performed using CFX manager (Biorad). Target genes were assayed with glyceraldehyde 3-phosphate dehydrogenase (*Gapdh*) for standardization. All reactions were performed in triplicate, and the average relative threshold cycle (ΔΔCt) from each sample was recorded for data analysis. The change in expression of each gene was determined relative to control mice after normalization to *Gapdh*.

### Evaluation of oxidative stress marker (Nitrotyrosine) by ELISA

RPE/choroid and retina from each sample were collected separately in phosphate buffered saline (PBS) with protease inhibitors (Thermo Fisher Scientific) and homogenized using manual homogenizer. The homogenate was centrifuged at 12,000 RPM for 30 minutes at 4°C, and the supernatant of tissue lysate was collected for ELISA. Each animal was assayed in triplicate and averaged to obtain a single data point; three animals per group were analyzed. The nitrotyrosine (3-nitrotyrosine, 3NT) competitive enzyme-linked immunosorbent assay (ELISA) (Abcam) was used to estimate the nitrotyrosine-modified protein levels from the retina/RPE/choroid sample, performed according to the manufacturer's protocol.

### Light and electron microscopy

After the overdose of sodium pentobarbital, mice were perfused with PBS containing 2% paraformaldehyde and 2.5% glutaraldehyde and the eyes were collected in the above mixture for light and electron microscopy. The eyes were processed according to the procedure described in our previous paper [35].

### Statistical analysis

The statistical software GraphPad Prism (version 5.0; Graph Pad Software, Inc., San Diego, CA, USA) was used to analyze the data. All reported P values were calculated using the 2-tailed Mann-Whitney test as indicated in the legends and a P value of <0.05 was considered significant. All data are represented as mean ± SEM unless otherwise indicated.

## Results

### Zeaxanthin supplementation leads to accumulation in liver and retina/RPE/choroid

**Fig 1:**
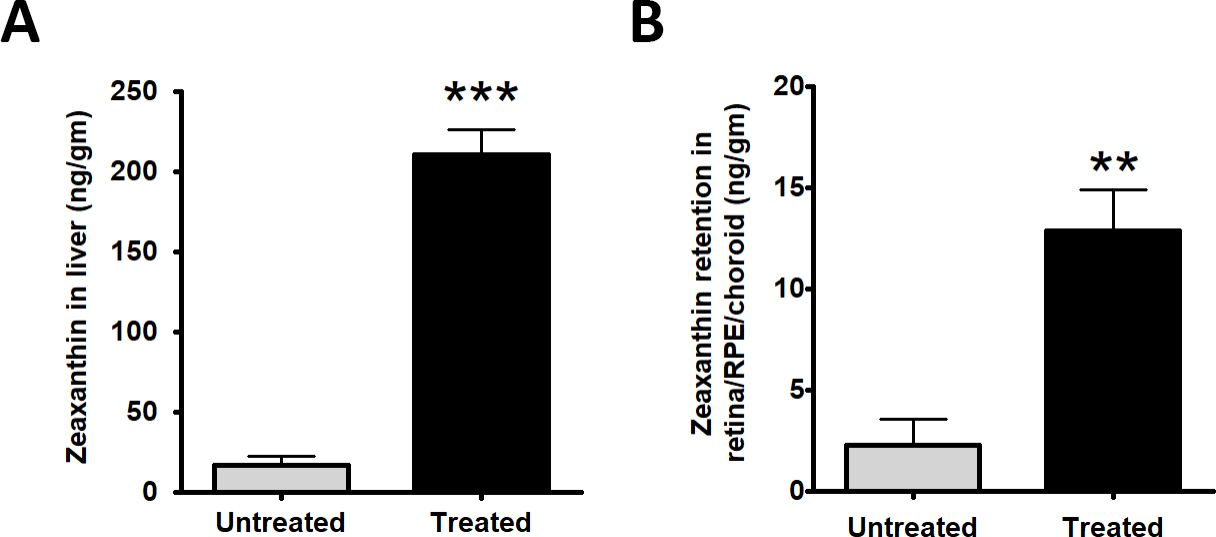
Zeaxanthin retention. The experimental group (n = 3) received daily gavage feeding of zeaxanthin beadlet at a dosage of 55-60mg/kg body weight for one month resulted in 12-fold retention of zeaxanthin in liver (n = 3) (A) and 5-fold retention in retina/RPE/choroid (n = 4) (B) compared to control untreated group (n = 3) on normal diet (Unpaired t-test, two tailed **P<0.01 and ***P <0.001).

After one month, the mean concentration of zeaxanthin in livers from experimental mice was 12 times higher than that in unsupplemented control mice (210.0 ± 15.7ng/gm (n = 3) vs 17 ± 5.44 ng/gm (n = 3); P<0.0010.003). Zeaxanthin concentration in the retina/RPE/choroid was 5-fold higher in supplemented mice (12.6 ± 1.8 ng/gm, n = 4) than in control mice (2.3 ± 0.8 ng/gm, n = 3); P<0.01 (Fig 1). Because the mouse retina expresses a carotenoid cleavage enzyme (β,β-carotene-9',10'-dioxygenase)[39], we suspect that zeaxanthin increased primarily in the RPE, but we have not yet validated this by analyzing zeaxanthin in dissected RPE.

### Zeaxanthin treatment changed antioxidant gene expression in RPE

**Fig 2:**
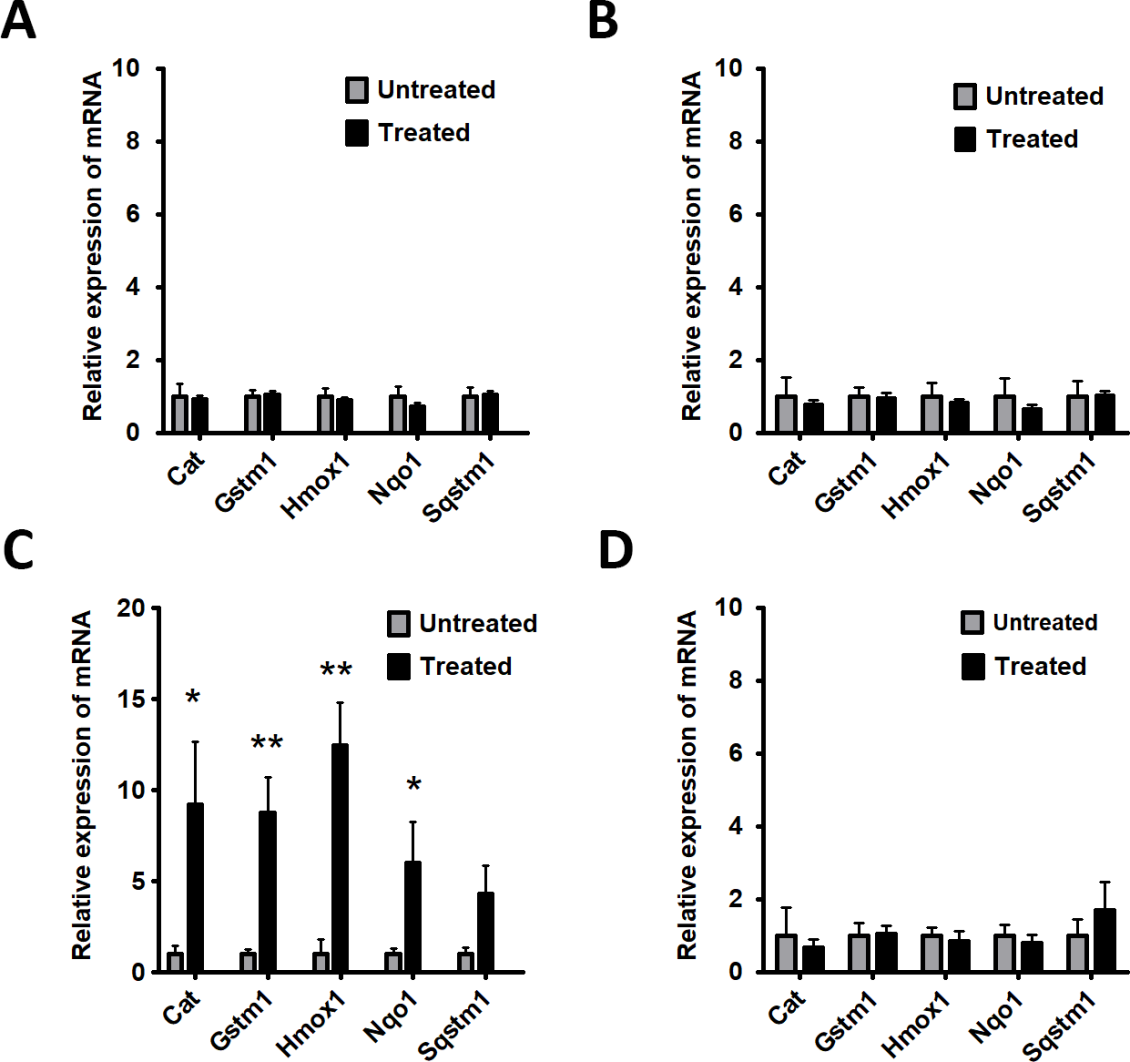
Zeaxanthin induces antioxidant response in the RPE. An experimental group (n = 5) receiving daily gavage feeding of zeaxanthin for one month did not exhibit induction of antioxidant genes such as Catalase (*Cat*), Glutathione S-Transferase Mu 1 (*Gstm1*), Heme Oxygenase 1 (*Hmox1*), NAD(P)H Quinone Dehydrogenase 1 (*Nqo1*), and Sequestosome 1 (*Sqstm1*) in RPE/choroid (A) or retina (B) compared to control group (n = 5). However, after four months of supplementation, the experimental group had greater mRNA expression of the antioxidant enzymes in the RPE/choroid (N = 5:P<0.05) than the control group (N = 5) (C); expression in the retina (D) was not affected by supplementation.

After one month and four months of gavage, the retina and RPE/choroid samples were harvested for quantitative real-time PCR analysis to measure levels of antioxidant response element regulated antioxidant transcripts, which are under the control of the Nrf2 (NFE2L2) transcriptional factor. No significant changes in antioxidant gene expression were seen after one-month of treatment (Fig 2A and 2B). At four months, the supplemented mice had more than 3-fold higher levels of Cat, *Gstm1*, *Hmox-1*, *Nqo-1 and Sqstm1* mRNA in the RPE/choroid (Fig 2C). There was no change in gene expression in the neural retina (Fig 2D).

### Reduction of protein nitration in RPE

**Fig 3:**
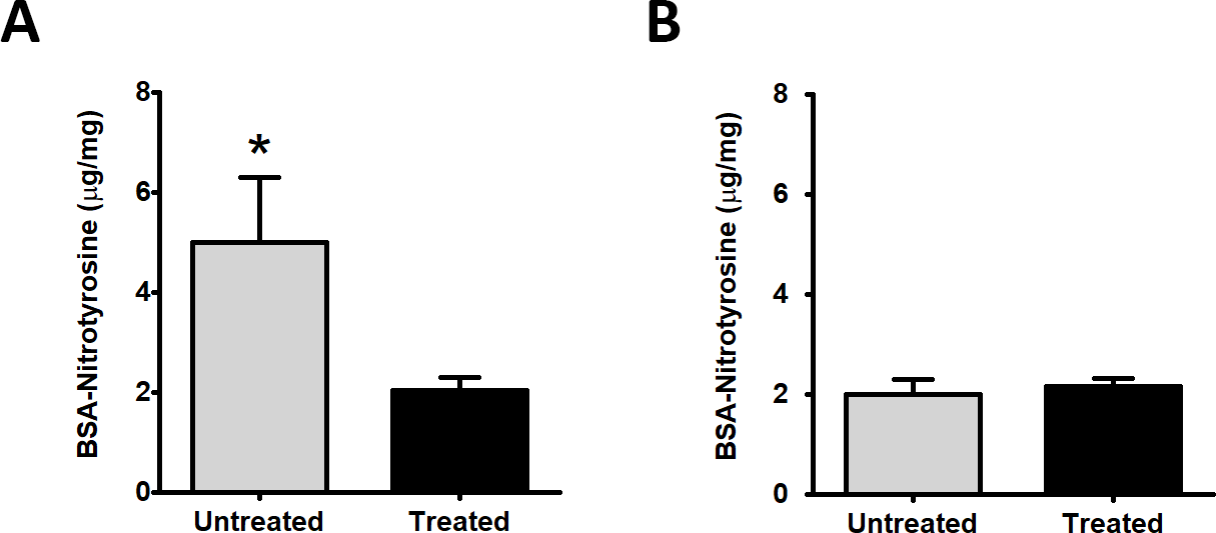
Reduced protein nitrosylation in the RPE following zeaxanthin treatment. In response to zeaxanthin treatment for 4 months, *Sod2*^*flox/flox*^*VMD2-cre mice* (n = 5) at 6 months of age showed a reduced level of nitrotyrosine in the RPE/choroid (A) but not in the retina where levels were already low (B) compared to eyes from untreated control *Sod2*^*flox/flox*^*VMD2-cre mice* (n = 5) by ELISA (*P < 0.5).

Tyrosine nitration arises from modification of tyrosine residues by peroxynitrite (ONOO-) formed from the reaction of superoxide (O_2_-^.^) with nitric oxide (NO). We previously reported that *Sod2* deletion in the RPE resulted in higher RPE concentrations of nitrotyrosine [37–38]. Zeaxanthin supplemented mice had 60% lower levels of nitrotyrosine (2.03±0.25 μg/mg, n = 6) in the RPE than the controls (5.0±1.3 μg/mg, n = 3; P = 0.015)(Fig 3A); but supplementation had no effect (P = 0.63) on nitrotyrosine concentration in the neural retina (2.15 ±0.3 μg/mg, n = 7 vs 2.0±0.16 μg/mg, n = 3) (Fig 3B).

### Improved c-wave ERG as a measure of RPE function

**Fig 4:**
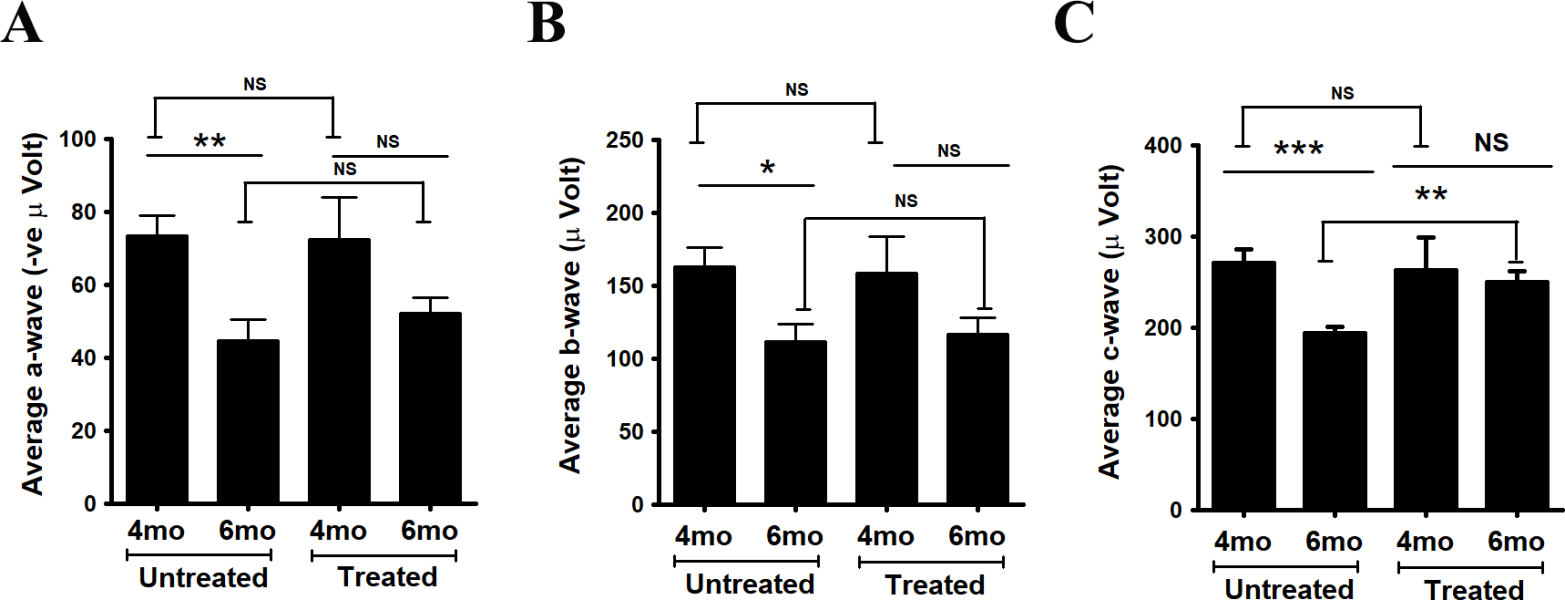
Improvement of RPE function detected by electroretinography. Dark-adapted electroretinogram (ERG) amplitudes measured at a light intensity of 20 cds/m^2^ at 4 months (4mo) and 6 months (6mo) of age after initiation of zeaxanthin treatment at 2 months of age. In the untreated group (n = 8), significant changes in (A) a-wave, (B) b-wave and (C) c-wave amplitudes were noticed at 6 months of age compared to 4 months of age. Daily gavage feeding of zeaxanthin did not result in any significant change in (A) a-wave and (B) b-wave amplitudes between control (n = 8) and zeaxanthin treated mice (n = 11) at 6 months (6mo) of age (4 months after onset of treatment). However, zeaxanthin fed *Sod2*^*flox/flox*^ *VMD2-cre* (C) mice had greater c-wave amplitudes at 6 months (6mo) of age (4 months after onset of treatment) than did the untreated *Sod2*^*flox/flox*^*VMD2-cre* mice at 6 months (6mo) of age following flashes of same light intensity (**P<0.01).

Electroretinography detects electrical responses of the retina to light flashes. The a-wave arises from photoreceptor cells, whereas the larger b-wave is the result of subsequent bipolar cell activity. The c-wave derives from the response of RPE to reduced potassium ion concentrations in the interphotoreceptor space during illumination [40]. As anticipated, there was a decrease in a-wave (from 73.4 ± 5.5, n = 8 to 44.8 ± 5.7, n = 8; P = 0.002) and b-wave (from 162.9 ± 13.4, n = 8 to 12.0 ± 11.6, n = 8; P = 0.01) amplitudes in control mice at 6 months of age compared to 4 months of age (Fig 4A and 4B). After 4 months of supplementation, comparison of ERG’s in supplemented and control mice at 6 months of age showed no significant difference in the a-wave (52.3 ± 4.1 n = 11 vs 44.8 ± 5.7, n = 8; P = 0.3) or b-wave (116.8 ± 11.4, n = 11 vs 112.0 ± 11.7, n = 8; P = 0.7) amplitudes. In our earlier characterization of this model, we found significant decline in the a-wave and b-wave responses in *Sod2*^*flox/flox*^*-VMD2-cre* mice only after six months of age [35]. In untreated mice, the c-wave amplitudes were lower at 6 months of age compared to 4 months of age. However, in the case of the c-wave we did observe benefit from zeaxanthin treatment. Even though the c-wave amplitude declined in the control group, the average c-wave amplitude (249.9 ± 12.1 μV, n = 11) in the supplemented mice did not decline between 4 months of age and 6 months of age. At both ages, the c-wave amplitudes in zeaxanthin supplemented mice were significantly higher than in control mice (Fig 4C). Representative ERG wave forms after 4 months of treatment are illustrated in S1 Fig.

### SD-OCT demonstrated improved RPE thickness

**Fig 5:**
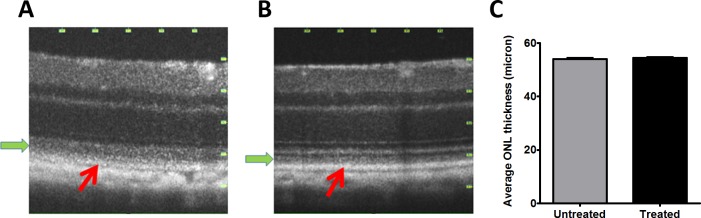
Zeaxanthin treatment improved structure of photoreceptor outer segments and the RPE. SD-OCT image of retinas from the zeaxanthin treated groups (n = 11) (B) after four months of treatment (6 months of age) shows preserved RPE layer and outer segment layer compared to eyes from untreated mice (n = 8) (A) of same age. We did not see any significant change in retinal outer nuclear layer (ONL) thickness (C) between control untreated and zeaxanthin-treated group. The red arrow indicates the RPE layer and the green arrow indicates the region of the photoreceptor outer segments.

Spectral domain optical coherence tomography (SD-OCT) is a reflectance technique used to measure the thickness of retinal layers and the accumulation of subretinal deposits [41]. In this mouse model of RPE oxidative stress, we have previously observed decrease in the thickness of the ONL at six months of age. After four months of treatment, SD-OCT examination detected no difference in ONL thickness in zeaxanthin-fed and control mice (Fig 5). However, comparison of OCT B-scans of zeaxanthin-treated and control mice revealed that the zeaxanthin-supplemented mice had sharper definition of the photoreceptor and RPE layers, more clearly resolved laminar structure, and reduced reflectance in outer nuclear layer (green arrows in Fig 5A and 5B).

### Improved RPE fine structure

**Fig 6:**
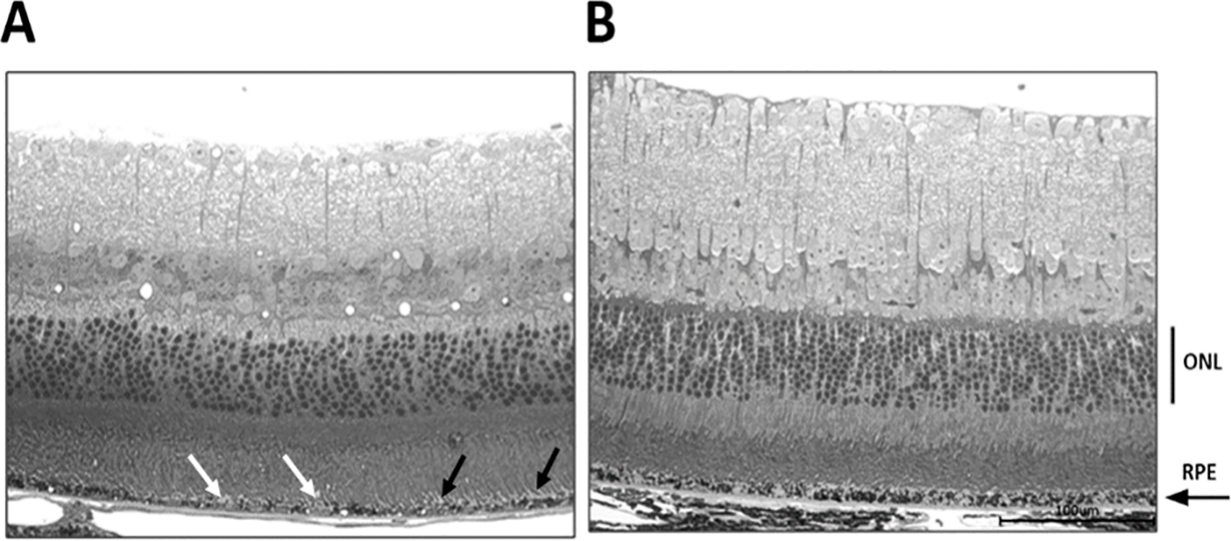
Light micrograph analysis. Preliminary data showed increased melanosomes in the RPE layer in the zeaxanthin treated eyes (n = 2) (B) compared to untreated eyes of same age (n = 2) (A). Both images are from the same location in the posterior retina of each eye. (scale bar 100μm). In A, black the arrow indicates a region in which outer segments of photoreceptors appear detached from the RPE and the white arrow head indicates a region of hypopigmentation in the RPE.

**Fig 7:**
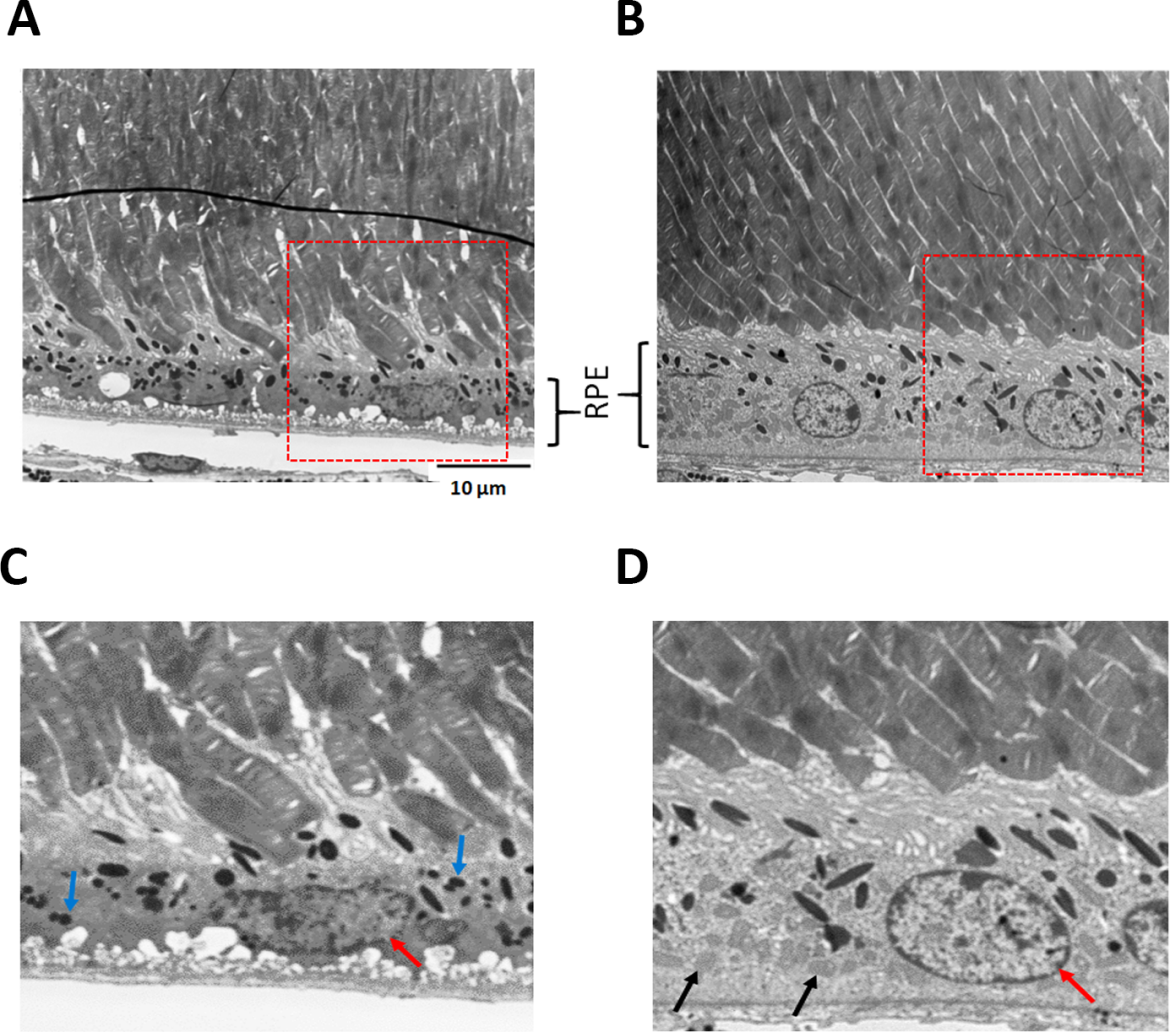
Improved ultrastructure of RPE. Ultrastructure analysis showed preservation of photoreceptors and RPE in the zeaxanthin treated eyes (n = 2) (B and D), whereas the eye from untreated mice (n = 2) (A and C) revealed broken tips of photoreceptors outer segments. Mitochondria were visible in the treated RPE (black arrows in D) but were sparse in the untreated eyes. The accumulation of lipofuscin granules (small black dots indicated by blue arrows in C) were reduced in treated eyes. RPE of the untreated eye had visible nuclear alterations such as nuclear pyknosis whereas the RPE cells in treated eyes had a normal, round nucleus (red arrow).

In light micrographs of 6-month old untreated mice, the RPE exhibited areas of reduced melanin (white arrow head) and interrupted contact with photoreceptor outer segments (black arrow) (Fig 6). Ultrastructural analysis of these untreated *Sod2*-deleted RPE revealed disorganized basal in-foldings, almost no mitochondria, and pyknotic nuclei (Fig 7A and 7D). Photoreceptor outer segments were poorly aligned and had broken tips as if phagocytosis of outer segments was impaired. In zeaxanthin-supplemented mice (Fig 7B and 7D), RPE had more organized and compact basal in-foldings, rounded nuclei with more dispersed chromatin, and many mitochondria at the basal surface, features typical of healthy RPE. Outer segments of photoreceptors were in parallel alignment, and no broken tips were apparent. Improved alignment of outer segments would improve their wave guiding properties and reduce the reflectance seen in the OCT images of unsupplemented mice.

## Discussion

Dietary supplementation with zeaxanthin protected the RPE from the impact of mitochondrial oxidative stress. Data presented here demonstrated that retention of zeaxanthin, presumably in the RPE, reduced oxidative stress as measured by nitrotyrosine and prevented the degenerative changes in RPE observed in the unsupplemented *Sod2* deficient mice.

Oxidative damage accumulating with age is a risk factor for age-related macular degeneration [42], and mitochondrial energy metabolism is a major source of reactive oxygen species. There are few animal studies, however, demonstrating the retention of carotenoids in retina, liver, and lens [28–30]. We, therefore, studied the effect of zeaxanthin supplementation in the *Sod2*^*flox/flox*^*VMD2-cre* mouse model of RPE-atrophy induced by chronic mitochondrial oxidative stress in RPE.

A recent clinical study involving combinational therapy showed that zeaxanthin did protect the macula in already diseased eyes [43]. These studies in advanced AMD were different from AREDS2 in that they used 10-fold higher (and putatively more effective) doses of zeaxanthin (20 mg vs 2). Zeaxanthin supplements reduced progression of CNV development in the fellow eye by 70–80%, and this was likely independent of the triple therapy to the contralateral eye consisting of: (1) reduced-fluence photodynamic therapy with verteporfin, (2) intravitreal bevacizumab and (3) intravitreal dexamethasone. It is reasonable to consider that the additional protection is associated with direct effects of zeaxanthin on the RPE. The rationale behind the use of the zeaxanthin alone in our experiment was to detect possible effects attributable only to carotenoid supplementation and its unique accumulation. In the future, it will be interesting to study the effects of lutein vs zeaxanthin supplementation or combinational effects of zeaxanthin and lutein in the *Sod2*^*flox/flox*^*VMD2-cre* mouse model. We would also specifically measure accumulation in isolated RPE.

Li and co-workers reported that wild-type C57BL/6 mice could not accumulate carotenoids in the retina due to the presence of highly active β,β-carotene-9',10'-dioxygenase (BCO2) enzyme which is inactive in the human retina [39]. Inactivation of this activity resulted in the accumulation of lutein and zeaxanthin in mouse retinas. Whether it was possible to bypass this activity and achieve significant delivery to the retina by supplementing with super physiological levels of zeaxanthin was not known; while our data indicate zeaxanthin was delivered to the RPE, we do not know whether the retina itself accumulated zeaxanthin. The zeaxanthin dose we employed was not derived from a dose response curve, but was based on the average American dietary intake. There is no basis on which one could argue that it is the optimal dose. Prophylactic treatments are sometimes given at doses above the normal physiological range, especially if it is known that the person is deficient in the normal uptake path. The rationale for a high dose of zeaxanthin was to provide enough zeaxanthin to saturate the activity of BCO2 and effectively deliver zeaxanthin to the mouse retina. In earlier work, zeaxanthin was added directly to mouse diet at 1 gm of zeaxanthin/kg or ~7.5ng/kg if fed to 30 gm BALB/c mice eating 4.5 grams of food per day [44]. We chose to give mice 1.15mg-1.5mg of zeaxanthin for 20gm to 25gm of mice. This ended up being of 56.3 mg to 60 mg of zeaxanthin per kg body weight of mice. The US Food and Drug Administration (Estimating the safe starting dose in clinical trials for therapeutics in adult healthy volunteers, U.S. Food and Drug Administration, Rockville, Maryland, USA) recommends that translation from human to animal dose should be based on body surface area, not on weight [45]. By this calculation 60 mg/kg in a mouse corresponds to a daily dosage of 4.9 mg/kg in a 60 kg adult human. While this is higher than the AEREDS2 recommendation, it is below the high dose tested in humans.

It would have been wise to measure the levels of zeaxanthin in the eye at multiple time points and, in particular, at 4 months. We chose one month, because we wanted to be sure that zeaxanthin was indeed accumulated despite the presence of β,β-carotene-9',10'-dioxygenase (BCO2) in the mouse retina. In other words, this time point was chosen to help us decide whether to continue the treatment. The livers accumulated around 12 times more zeaxanthin than control livers after one month. It is possible, and indeed likely, that zeaxanthin continued to accumulate in the retina/RPE/choroid, but we did not measure accumulation after a longer interval, and this is a shortcoming of our experiments. Uptake, metabolism, and stabilization of xanthophyll carotenoids in the retina are thought to be mediated by specific xanthophyll-binding proteins (XBPs). The Pi isoform of human glutathione S-transferase (GSTP1) is a zeaxanthin-binding protein whose enzyme activity is increased when zeaxanthin is bound [46]. In our future studies; we aim to investigate GSTP1 protein expression in the RPE/choroid and retina in response to zeaxanthin accumulation in the RPE.

The protection of structure and function of RPE raised questions about the mechanism of protection. Zeaxanthin effectively protected against tert-butyl hydroperoxide-induced mitochondrial dysfunction and apoptosis in RPE cells through NF-E2-related factor 2 (NRF2) activation [27]. Zeaxanthin was further reported to protect a rat model of oxidative damage by inducing NRF2 and HO-1 [47]. In *Sod2*^*flox/flox*^*VMD2-cre* mice we expected greater oxidative stress in RPE since an essential antioxidant enzyme was deleted in the RPE. Our observation that zeaxanthin increased RPE expression of NRF2 -regulated enzymes, including catalase, NAD(P)H quinone dehydrogenase 1and heme oxygenase 1 confirmed the induction of antioxidant enzyme mechanism *in vivo*. The mu class of glutathione S-transferase (GSTM1) enzyme functions in the detoxification of electrophilic compounds, and products of oxidative stress, by conjugation with glutathione [48]. Dietary supplementation of zeaxanthin also increased GSTM1 expression in *Sod2*^*flox/flox*^*VMD2-cre* mice. Since the ARE genes are regulated at the stage of transcription, we presume that the protein levels also increased, but we did not confirm this using immunological methods. Zou *et al*. also showed that oral supplementation of zeaxanthin induces Nrf2-mediated phase II enzymes *in vitro* and *in vivo* [27]. Not having the protein data to support increased antioxidant defense by RPE is a weakness in the current study and it will be explored in future studies.

Oxidative stress marker nitrotyrosine is produced by the modification of protein tyrosine residues by peroxynitrite generated from the reaction of nitric oxide (NO) and superoxide. Increased nitrotyrosine level in the retina was reported in the animal model of photoreceptor oxidative damage [49] and aging human Bruch’s membrane [50]. We have reported significant elevation of nitrotyrosine in *Sod2*^*flox/flox*^*VMD2-cre* mouse by 2 months of age [35] and that its level was decreased in response to systemic treatment with a 5HT1a agonist [38]. Dietary supplementation of zeaxanthin led to significant reduction of nitrotyrosine level in RPE/choroid (Fig 3). We did not find significant changes in nitrotyrosine levels in the retina of treated and untreated mice, but nitrotyrosine levels were already low. The fact that the nitrotyrosine levels were not affected in the retina indicates that the oxidative damage in the retina is due to local generation of peroxynitrite, and further supports the view that zeaxanthin did not accumulate in the retina of these mice. Free radicals are too short-lived to move from the RPE across the subretinal space to the retina.

Supplementation with zeaxanthin did not support improvement in photoreceptor layer thickness by SD-OCT, despite a 5-fold increase in zeaxanthin retention in the retina/RPE/choroid after one month of supplementation. We anticipated that increased zeaxanthin retention in RPE would benefit retinal function as detected by ERG a- or b-wave responses after four months of treatment. However, despite the preservation of RPE function, based on the c-wave response and improved RPE ultrastructure in response to zeaxanthin treatment, we did not see preservation of neural retina structure and function. In this model, initial RPE dysfunction affects neural retina function secondarily. The fact that c-waves in 6 month old treated mice are same as in 4 month old mice suggests that the implied retention of RPE functionality did not benefit neural retina function. We suspect that the presence of BCO2 enzyme activity in the retina prevented zeaxanthin accumulation in the retina, thus interrupting any antioxidant protection or induction of antioxidant enzymes from RPE. However, for practical reasons, we stopped our analysis before we expected the statistically significant reduction in ERG a-wave and b-wave amplitudes or ONL thickness that we had previously shown [35]. The experiments in Figs 6 and 7 had a very low n n = 2, and therefore are presented solely as preliminary data encouraging the future work necessary to reach concrete conclusions. Protection of the RPE in our experiments implies that zeaxanthin did accumulate in the RPE as confirmed by the induction of Nrf2-regulated enzymes, the preservation of RPE structural integrity, and the protection of RPE function as indicated by the preserved c-wave amplitude (Fig 4) and by the preserved ultrastructure of the RPE (Fig 7).

## Conclusions

In our experiments, dietary zeaxanthin induced Nrf2-dependent antioxidant enzymes, thus protecting RPE from oxidative induced damage and substantially preserved RPE structure and function. Because oxidative stress in the RPE is implicated in age-related macular degeneration, we believe that daily intake of zeaxanthin may reduce the risk for development of AMD. Further studies in BCO2 knockout mice are needed to assess whether elevating retinal zeaxanthin can retard RPE and photoreceptor atrophy. Additional questions to be addressed include whether zeaxanthin supplementation could slow disease progression after retinal and/or RPE degeneration is already initiated and whether this intervention could be useful in diseases in which the primary insult does not involve RPE oxidative stress [51].

## Supporting information

S1 Fig ERG wave forms. Representative ERG wave forms in dark adapted untreated (red line) and zeaxanthin treated (black line) *Sod2*^*flox/flox*^*VMD2-cre* mice taken after four months of treatment. Flash intensity was 20 cds/m^2^.
(TIF)

S1 Table Primers of genes analyzed by Real time PCR. (DOCX)

## Abbreviations

AMD: age-related macular degeneration
SOD: superoxide dismutase
RPE: retinal pigmented epithelium
VEGF: vascular endothelial growth factor
AREDS: Age-Related Eye Disease Study
GSTP1: *Pi* isoform of human glutathione S-transferase
ERG: electroretinography
SD-OCT: spectral domain optical coherence tomography
ONL: outer nuclear layer containing photoreceptor cell bodies
ARE: anti-oxidant response element
CAT: catalase
ELISA: enzyme-linked immunosorbent assay
BCO2: β,β-carotene-9',10'-dioxygenase
NRF2: NF-E2-related factor 2
