# Supplementary figures and images for: Daily zeaxanthin supplementation prevents atrophy of the retinal pigment epithelium (RPE) in a mouse model of mitochondrial oxidative stress
Source: PLoS One. 2018 Sep 28;13(9):e0203816. doi: 10.1371/journal.pone.0203816 (PMC6161850; doi:10.1371/journal.pone.0203816)

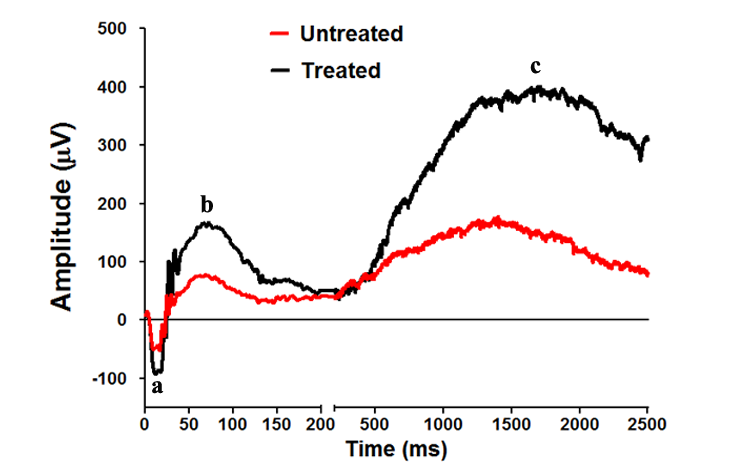

Supplement: S1 Fig — Representative ERG wave forms in dark adapted untreated (red line) and zeaxanthin treated (black line) Sod2flox/floxVMD2-cre mice taken after four months of treatment. Flash intensity was 20 cds/m2. (TIF) [file pone.0203816.s001.tif]
