# Supplementary material for: Daily zeaxanthin supplementation prevents atrophy of the retinal pigment epithelium (RPE) in a mouse model of mitochondrial oxidative stress
Source: PLoS One. 2018 Sep 28;13(9):e0203816. doi: 10.1371/journal.pone.0203816 (PMC6161850; doi:10.1371/journal.pone.0203816)
Supplement: S1 Table — (DOCX) [file pone.0203816.s002.docx]

**S1 Table**. **Primers of genes analyzed by Real time PCR.**

| **GENE NAME** | **NCBI ID** | **FWD PRIMER (5’-3’)** | **REVERSE PRIMER (5’-3’)** | **AMPLICON SIZE (bp)** |
| --- | --- | --- | --- | --- |
| *Cat* | NM_009804.2 | CGCAATCCTACACCATGTCG | AGTATCCAAAAGCACCTGCTCC | 267 |
| *Gstm1* | NM_010358.5 | GGGATACTGGAACGTCCGC | GCTCTGGGTGATCTTGTGTGA | 205 |
| *Hmox-1* | NM_010442.2 | AGCCCCACCAAGTTCAAACA | GCAGTATCTTGCACCAGGCT | 224 |
| *Nqo-1* | NM_008706.5 | CGACAACGGTCCTTTCCAGA | CCAGACGGTTTCCAGACGTT | 250 |
| *Sqstm1* | NM_011018.2 | GGAAGCTGCCCTATACCCAC | GCTTGGCCACAGCACTATCA | 207 |
| *Gapdh* | NM_008084.2 | TCTCCTGCGACTTCAACAGC | GATAGGGCCTCTCTTGCTCAG | 217 |
